# Supplementary material for: Comparison of survival in patients with low vs. intermediate prostate-specific antigen concentrations and development of a nomogram: a surveillance, epidemiology and end results program database study with external validation on a Chinese cohort
Source: PeerJ. 2025 Aug 4;13:e19823. doi: 10.7717/peerj.19823 (PMC12330820; doi:10.7717/peerj.19823)
Supplement: Supplemental Information 1 [file peerj-13-19823-s001.docx]

| Variable | T1 | | T2 | | T3 | | T4 | |
| --- | --- | --- | --- | --- | --- | --- | --- | --- |
|  | PSA≤4.0 | PSA 4-10 | PSA≤4.0 | PSA 4-10 | PSA≤4.0 | PSA 4-10 | PSA≤4.0 | PSA 4-10 |
| N, (%) | 13142 (14.09) | 80103 (85.91) | 19934 (21.64) | 72175 (78.36) | 2242 (12.82) | 15242 (87.18) | 29 (14.50) | 171 (85.50) |
| Age, N(%) |  |  |  |  |  |  |  |  |
| <65 | 6450 (49.08) | 33380 (41.67) | 12213 (61.27) | 40690 (56.38) | 1311 (58.47) | 8607 (56.47) | 16 (55.17) | 95 (55.56) |
| 65-69 | 2877 (21.89) | 21639 (27.01) | 3973 (19.93) | 17520 (24.27) | 567 (25.29) | 4129 (27.09) | 1 (3.45) | 31 (18.13) |
| 70-74 | 2139 (16.28) | 14918 (18.62) | 2390 (11.99) | 8868 (12.29) | 275 (12.27) | 1957 (12.84) | 5 (17.24) | 19 (11.11) |
| 75-79 | 1140 (8.67) | 7609 (9.50) | 974 (4.89) | 3682 (5.10) | 73 (3.26) | 453 (2.97) | 2 (6.90) | 15 (8.77) |
| 80-84 | 376 (2.86) | 2108 (2.63) | 306 (1.54) | 1149 (1.59) | 11 (0.49) | 85 (0.56) | 2 (6.90) | 8 (4.68) |
| ≥85 | 160 (1.22) | 449 (0.56) | 78 (0.39) | 266 (0.37) | 5 (0.22) | 11 (0.07) | 3 (10.34) | 3 (1.75) |
| Race, N(%) |  |  |  |  |  |  |  |  |
| White | 10253 (78.02) | 59927 (74.81) | 16762 (84.09) | 57828 (80.12) | 1949 (86.93) | 12593 (82.62) | 21 (72.41) | 129 (75.44) |
| Black | 2180 (16.59) | 14703 (18.36) | 2177 (10.92) | 9723 (13.47) | 197 (8.79) | 1718 (11.27) | 7 (24.14) | 32 (18.71) |
| Other | 709 (5.39) | 5473 (6.83) | 995 (4.99) | 4624 (6.41) | 96 (4.28) | 931 (6.11) | 1 (3.45) | 10 (5.85) |
| Stage N, N(%) |  |  |  |  |  |  |  |  |
| N0 | 13133 (99.93) | 80028 (99.91) | 19892 (99.79) | 71869 (99.58) | 2144 (95.63) | 14474 (94.96) | 23 (79.31) | 144 (84.21) |
| N1 | 9 (0.07) | 75 (0.09) | 42 (0.21) | 306 (0.42) | 98 (4.37) | 768 (5.04) | 6 (20.69) | 27 (15.79) |
| Stage M, N(%) |  |  |  |  |  |  |  |  |
| M0 | 13120 (99.83) | 79955 (99.82) | 19900 (99.83) | 72056 (99.84) | 2233 (99.60) | 15187 (99.64) | 26 (89.66) | 155 (90.64) |
| M1 | 22 (0.17) | 148 (0.18) | 34 (0.17) | 119 (0.16) | 9 (0.40) | 55 (0.36) | 3 (10.34) | 16 (9.36) |
| Local treatment, N(%) |  |  |  |  |  |  |  |  |
| Yes | 5022 (38.21) | 40192 (50.18) | 16018 (80.36) | 62322 (86.35) | 2218 (98.93) | 15083 (98.96) | 24 (82.76) | 134 (78.36) |
| No | 8120 (61.79) | 39911 (49.82) | 3916 (19.64) | 9853 (13.65) | 24 (1.07) | 159 (1.04) | 5 (17.24) | 37 (21.64) |

Table S1. Baseline characteristics of patients with Gleason Score 6-7 according to T-category in SEER program.
