# Supplementary material for: Comparison of survival in patients with low vs. intermediate prostate-specific antigen concentrations and development of a nomogram: a surveillance, epidemiology and end results program database study with external validation on a Chinese cohort
Source: PeerJ. 2025 Aug 4;13:e19823. doi: 10.7717/peerj.19823 (PMC12330820; doi:10.7717/peerj.19823)
Supplement: Supplemental Information 2 [file peerj-13-19823-s002.docx]

| Variable | T1 | | T2 | | T3 | | T4 | |
| --- | --- | --- | --- | --- | --- | --- | --- | --- |
|  | PSA≤4.0 | PSA 4-10 | PSA≤4.0 | PSA 4-10 | PSA≤4.0 | PSA 4-10 | PSA≤4.0 | PSA 4-10 |
| N, (%) | 987 (10.54) | 8375 (89.46) | 1882 (14.93) | 10726(85.07) | 1150 (14.26) | 6916 (85.74) | 170 (35.41) | 310 (64.59) |
| Age, N(%) |  |  |  |  |  |  |  |  |
| <65 | 213 (21.58) | 1854 (22.14) | 602 (31.99) | 3731 (34.78) | 480 (41.74) | 2968 (42.91) | 42 (24.71) | 103 (33.23) |
| 65-69 | 173 (17.53) | 1860 (22.21) | 444 (23.59) | 2710 (25.27) | 314 (27.30) | 1990 (28.77) | 41 (24.12) | 74 (23.87) |
| 70-74 | 231 (23.40) | 2073 (24.75) | 391 (20.78) | 2166 (20.19) | 214 (18.61) | 1305 (18.87) | 30 (17.65) | 50 (16.13) |
| 75-79 | 197 (19.96) | 1655 (19.76) | 271 (14.40) | 1322 (12.33) | 94 (8.17) | 473 (6.84) | 23 (13.53) | 32 (10.32) |
| 80-84 | 112 (11.35) | 698 (8.33) | 126 (6.70) | 604 (5.63) | 38 (3.30) | 141 (2.04) | 14 (8.24) | 28 (9.03) |
| ≥85 | 61 (6.18) | 235 (2.81) | 48 (2.55) | 193 (1.80) | 10 (0.87) | 39 (0.56) | 20 (11.76) | 23 (7.42) |
| Race, N(%) |  |  |  |  |  |  |  |  |
| White | 773 (78.32) | 6120 (73.07) | 1583 (84.11) | 8471 (78.98) | 1026 (89.22) | 5736 (82.94) | 148 (87.06) | 260 (83.87) |
| Black | 153 (15.50) | 1603 (19.14) | 188 (9.99) | 1356 (12.64) | 71 (6.17) | 652 (9.43) | 10 (5.88) | 25 (8.06) |
| Other | 61 (6.18) | 652 (7.79) | 111 (5.90) | 899 (8.38) | 53 (4.61) | 528 (7.63) | 12 (7.06) | 25 (8.06) |
| Stage N, N(%) |  |  |  |  |  |  |  |  |
| N0 | 944 (95.64) | 8210 (98.03) | 1822 (96.81) | 10378 (96.76) | 946 (82.26) | 5748 (83.11) | 100 (58.82) | 176 (56.77) |
| N1 | 43 (4.36) | 165 (1.97) | 60 (3.19) | 348 (3.24) | 204 (17.74) | 1168 (16.89) | 70 (41.18) | 134 (43.23) |
| Stage M, N(%) |  |  |  |  |  |  |  |  |
| M0 | 896 (90.78) | 8067 (96.32) | 1797 (95.48) | 10412 (97.07) | 1097 (95.39) | 6698 (96.85) | 106 (62.35) | 222 (71.61) |
| M1 | 91 (9.22) | 308 (3.68) | 85 (4.52) | 314 (2.93) | 53 (4.61) | 218 (3.15) | 64 (37.65) | 88 (28.39) |
| Local treatment, N(%) |  |  |  |  |  |  |  |  |
| Yes | 633 (64.13) | 6291 (75.12) | 1594 (84.70) | 9411 (87.74) | 1084 (94.26) | 6665 (96.37) | 95 (55.88) | 197 (63.55) |
| No | 354 (35.87) | 2084 (24.88) | 288 (15.30) | 1315 (12.26) | 66 (5.74) | 251 (3.63) | 75 (44.12) | 113 (36.45) |

Table S2. Baseline characteristics of patients with Gleason Score 8-10 according to T-category in SEER program.
