# Supplementary material for: Comparison of survival in patients with low vs. intermediate prostate-specific antigen concentrations and development of a nomogram: a surveillance, epidemiology and end results program database study with external validation on a Chinese cohort
Source: PeerJ. 2025 Aug 4;13:e19823. doi: 10.7717/peerj.19823 (PMC12330820; doi:10.7717/peerj.19823)
Supplement: Supplemental Information 3 [file peerj-13-19823-s003.docx]

| Variable | T1 | | T2 | | T3 | | T4 | |
| --- | --- | --- | --- | --- | --- | --- | --- | --- |
|  | HR (95% CI) | P | HR (95% CI) | P | HR (95% CI) | P | HR (95% CI) | P |
| Age |  |  |  |  |  |  |  |  |
| <65 | 1 | - | 1 | - | 1 | - | 1 | - |
| 65-69 | 1.76 (1.65 - 1.88) | <0.0001 | 1.93 (1.80 - 2.08) | <0.0001 | 1.76 (1.50 - 2.08) | <0.0001 | 1.63 (0.60 - 4.42) | 0.3331 |
| 70-74 | 2.48 (2.32 - 2.64) | <0.0001 | 3.17 (2.94 - 3.43) | <0.0001 | 2.17 (1.79 - 2.65) | <0.0001 | 3.69 (1.45 - 9.35) | 0.006 |
| 75-79 | 3.87 (3.61 - 4.14) | <0.0001 | 5.52 (5.07 - 6.01) | <0.0001 | 4.23 (3.21 - 5.57) | <0.0001 | 3.88 (1.44 - 10.44) | 0.0072 |
| 80-84 | 6.55 (5.99 - 7.16) | <0.0001 | 8.63 (7.71 - 9.65) | <0.0001 | 9.66 (6.32 - 14.77) | <0.0001 | 7.24 (2.24 - 23.39) | 0.0009 |
| ≥85 | 12.18 (10.68 - 13.89) | <0.0001 | 14.87 (12.53 - 17.65) | <0.0001 | 26.61 (12.2 - 58.05) | <0.0001 | 13.1 (3.46 - 49.61) | 0.0002 |
| Race |  |  |  |  |  |  |  |  |
| White | 1 | - | 1 | - | 1 | - | 1 | - |
| Black | 1.44 (1.37 - 1.52) | <0.0001 | 1.42 (1.31 - 1.53) | <0.0001 | 1.44 (1.17 - 1.76) | 0.0005 | 2.57 (1.08 - 6.13) | 0.0332 |
| Other | 0.55 (0.49 - 0.62) | <0.0001 | 0.52 (0.45 - 0.60) | <0.0001 | 0.62 (0.42 - 0.90) | 0.0120 | 2.09 (0.64 - 6.83) | 0.2248 |
| PSA level |  |  |  |  |  |  |  |  |
| 4.1-10.0 | 1 | - | 1 | - | 1 | - | 1 | - |
| ≤4.0 | 0.91 (0.85 - 0.97) | 0.0036 | 1.04 (0.98 - 1.11) | 0.1988 | 1.10 (0.91 - 1.33) | 0.3453 | 1.38 (0.61 - 3.12) | 0.4354 |
| Stage N |  |  |  |  |  |  |  |  |
| N0 | 1 | - | 1 | - | 1 | - | 1 | - |
| N1 | 2.78 (1.79 - 4.33) | <0.0001 | 1.99 (1.43 - 2.77) | <0.0001 | 1.75 (1.35 - 2.29) | <0.0001 | 3.52 (1.69 - 7.31) | 0.0007 |
| Stage M |  |  |  |  |  |  |  |  |
| M0 | 1 | - | 1 | - | 1 | - | 1 | - |
| M1 | 3.35 (2.54 - 4.43) | <0.0001 | 4.78 (3.65 - 6.25) | <0.0001 | 3.97 (2.28 - 6.91) | <0.0001 | 4.84 (2.18 - 10.77) | 0.0001 |
| Local treatment |  |  |  |  |  |  |  |  |
| Yes | 1 | - | 1 | - | 1 | - | 1 | - |
| No | 1.15 (1.10 - 1.21) | <0.0001 | 1.62 (1.53 - 1.73) | <0.0001 | 1.70 (1.09 - 2.66) | 0.0196 | 3.05 (1.56 - 5.98) | 0.0012 |

Table S3. The outcomes of multivariate Cox regression analyses according to T-category in the GS 6–7.
