# Supplementary material for: Comparison of survival in patients with low vs. intermediate prostate-specific antigen concentrations and development of a nomogram: a surveillance, epidemiology and end results program database study with external validation on a Chinese cohort
Source: PeerJ. 2025 Aug 4;13:e19823. doi: 10.7717/peerj.19823 (PMC12330820; doi:10.7717/peerj.19823)
Supplement: Supplemental Information 4 [file peerj-13-19823-s004.docx]

| Variable | T1 | | T2 | | T3 | | T4 | |
| --- | --- | --- | --- | --- | --- | --- | --- | --- |
|  | HR (95% CI) | P | HR (95% CI) | P | HR (95% CI) | P | HR (95% CI) | P |
| Age |  |  |  |  |  |  |  |  |
| <65 | 1 | - | 1 | - | 1 | - | 1 | - |
| 65-69 | 1.31 (1.10-1.56) | 0.0022 | 1.53 (1.31 - 1.79) | <0.0001 | 0.95 (0.8 - 1.13) | 0.5710 | 1.45 (0.97 - 2.19) | 0.0731 |
| 70-74 | 1.84 (1.57 - 2.16) | <0.0001 | 2.28 (1.96 - 2.66) | <0.0001 | 1.24 (1.03 - 1.50) | 0.0231 | 1.69 (1.10 - 2.6) | 0.0165 |
| 75-79 | 2.08 (1.77 - 2.45) | <0.0001 | 3.32 (2.85 - 3.86) | <0.0001 | 1.78 (1.41 - 2.24) | <0.0001 | 1.93 (1.20 - 3.11) | 0.0064 |
| 80-84 | 3.21 (2.7 - 3.83) | <0.0001 | 4.48 (3.76 - 5.33) | <0.0001 | 3.75 (2.84 - 4.96) | <0.0001 | 2.93 (1.78 - 4.84) | <0.0001 |
| ≥85 | 5.76 (4.7 - 7.07) | <0.0001 | 7.53 (6.07 - 9.33) | <0.0001 | 3.36 (2.15 - 5.24) | <0.0001 | 3.65 (2.27 - 5.88) | <0.0001 |
| Race |  |  |  |  |  |  |  |  |
| White | 1 | - | 1 | - | 1 | - | 1 | - |
| Black | 1.10 (0.97 - 1.24) | 0.1538 | 1.04 (0.89 - 1.22) | 0.6452 | 0.97 (0.76 - 1.24) | 0.8167 | 1.09 (0.63 - 1.88) | 0.7631 |
| Other | 0.61 (0.49 - 0.76) | <0.0001 | 0.46 (0.36 - 0.58) | <0.0001 | 0.68 (0.50 - 0.92) | 0.014 | 0.68 (0.38 - 1.2) | 0.1816 |
| PSA level |  |  |  |  |  |  |  |  |
| 4.1-10.0 | 1 | - | 1 | - | 1 | - | 1 | - |
| ≤4.0 | 1.18 (1.04 - 1.35) | 0.0136 | 1.20 (1.07 - 1.35) | 0.0026 | 1.20 (1.01 - 1.42) | 0.0331 | 1.35 (1.03 - 1.78) | 0.0294 |
| Stage N |  |  |  |  |  |  |  |  |
| N0 | 1 | - | 1 | - | 1 | - | 1 | - |
| N1 | 1.17 (0.92 - 1.5) | 0.2024 | 1.38 (1.11 - 1.71) | 0.0032 | 1.47 (1.25 - 1.73) | <0.0001 | 1.23 (0.93 - 1.63) | 0.1435 |
| Stage M |  |  |  |  |  |  |  |  |
| M0 | 1 | - | 1 | - | 1 | - | 1 | - |
| M1 | 4.13 (3.53 - 4.84) | <0.0001 | 3.49 (2.95 - 4.13) | <0.0001 | 3.07 (2.38 - 3.96) | <0.0001 | 2.76 (2.03 - 3.75) | <0.0001 |
| Local treatment |  |  |  |  |  |  |  |  |
| Yes | 1 | - | 1 | - | 1 | - | 1 | - |
| No | 2.00 (1.81 - 2.22) | <0.0001 | 2.22 (1.98 - 2.48) | <0.0001 | 2.80 (2.19 - 3.59) | <0.0001 | 1.62 (1.20 - 2.18) | 0.0016 |

Table S4. The outcomes of multivariate Cox regression analyses according to T-category in the GS 8-10.
