# Supplementary figures and images for: Comparison of survival in patients with low vs. intermediate prostate-specific antigen concentrations and development of a nomogram: a surveillance, epidemiology and end results program database study with external validation on a Chinese cohort
Source: PeerJ. 2025 Aug 4;13:e19823. doi: 10.7717/peerj.19823 (PMC12330820; doi:10.7717/peerj.19823)

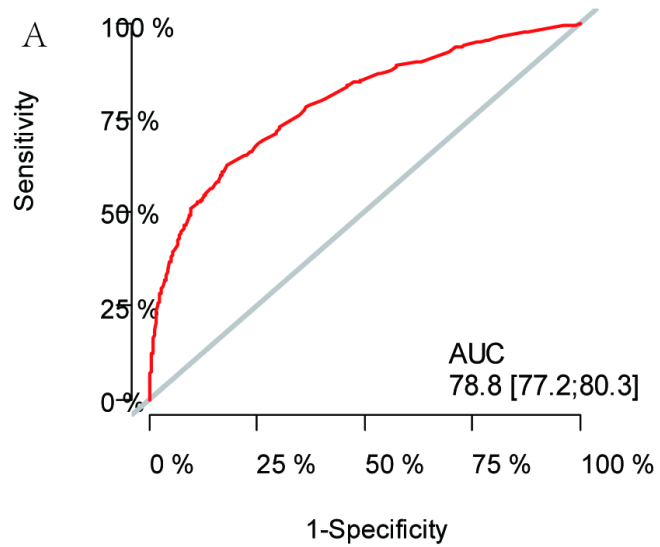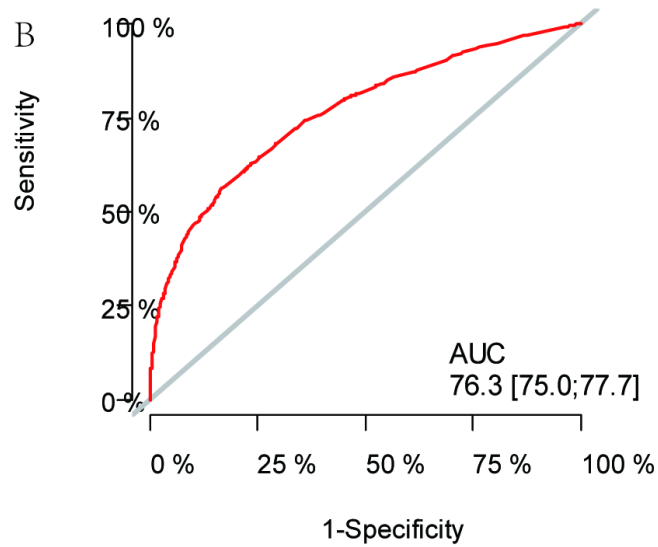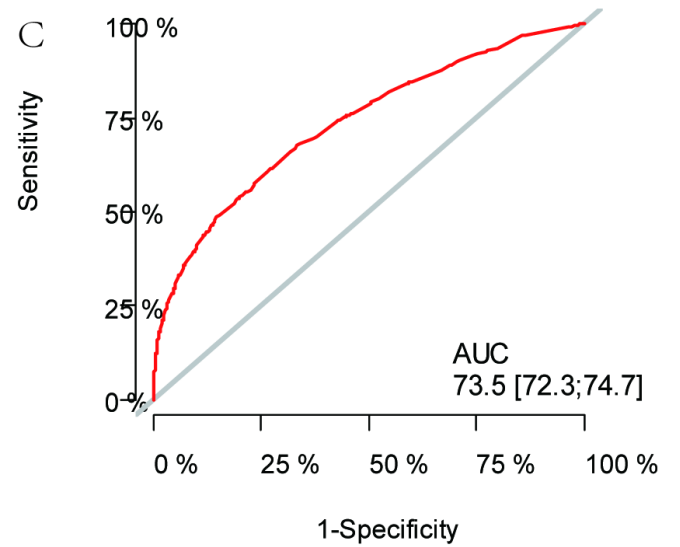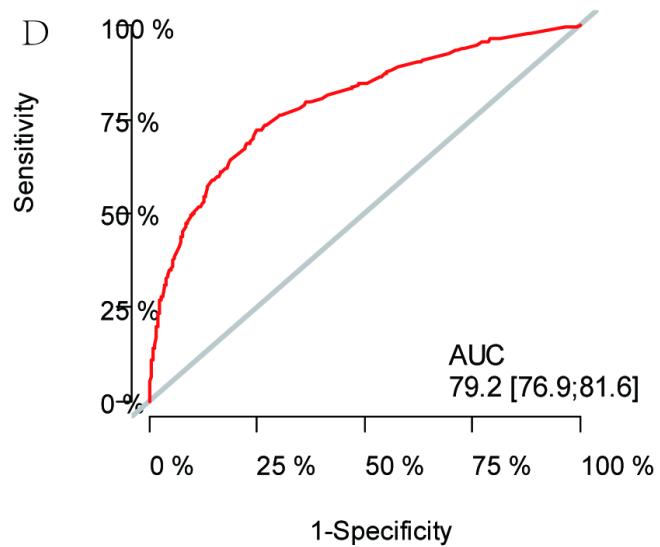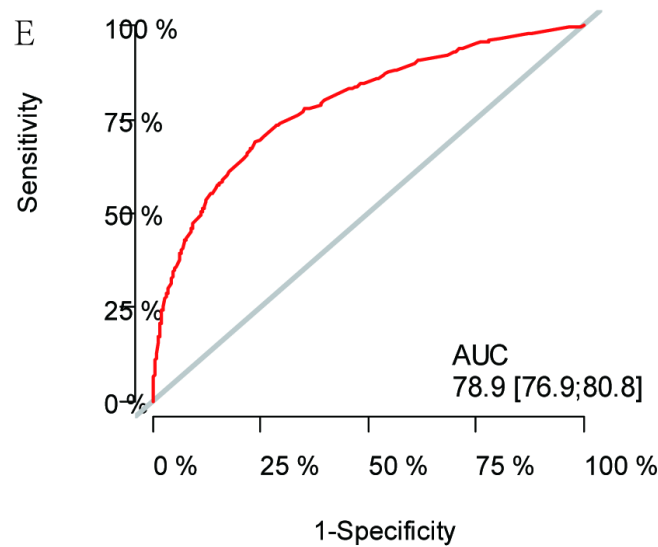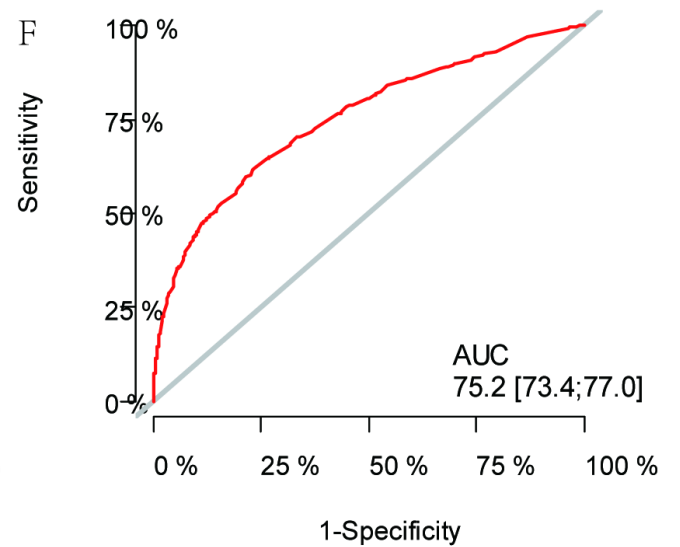

Supplement: Supplemental Information 5 [file peerj-13-19823-s005.pdf]

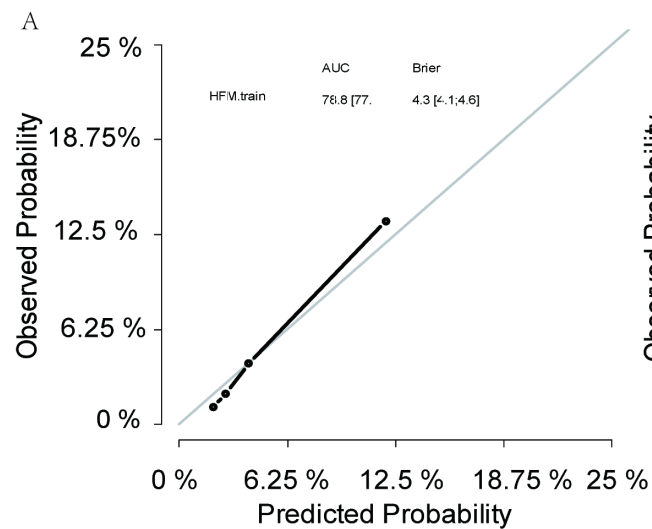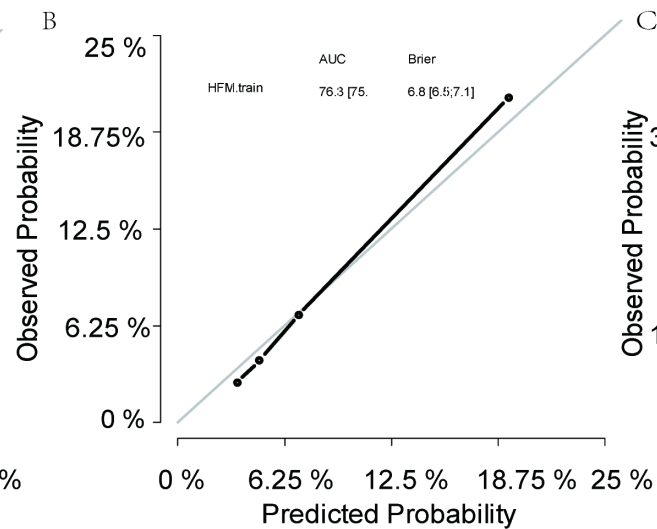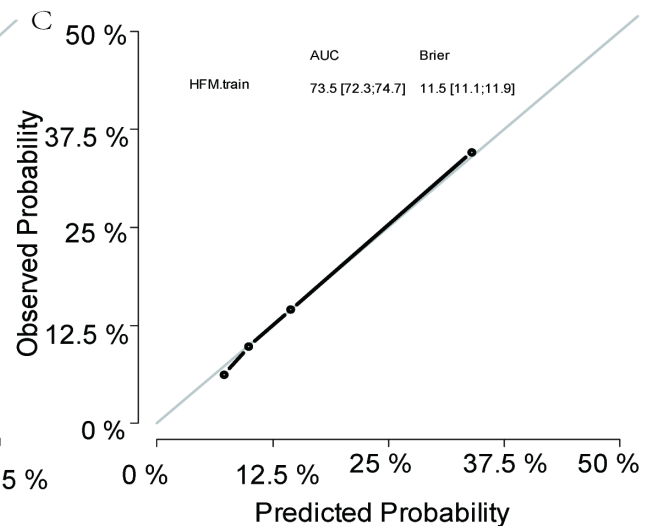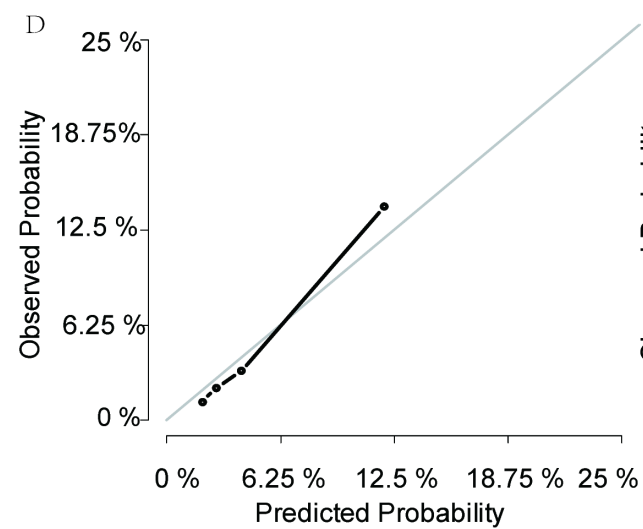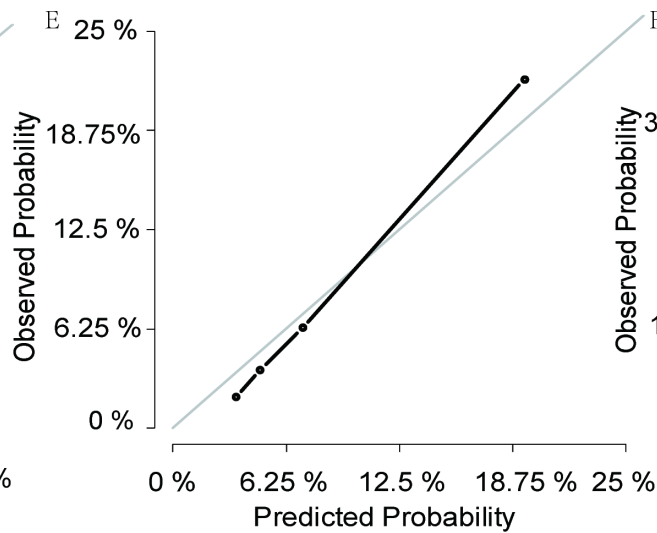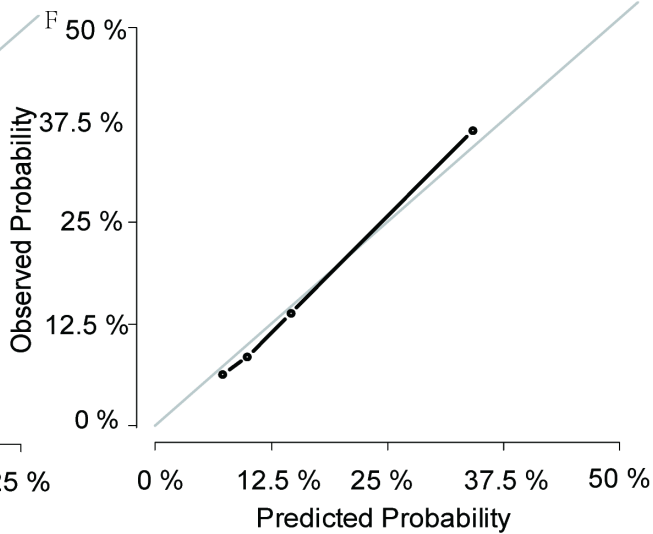

Supplement: Supplemental Information 6 [file peerj-13-19823-s006.pdf]

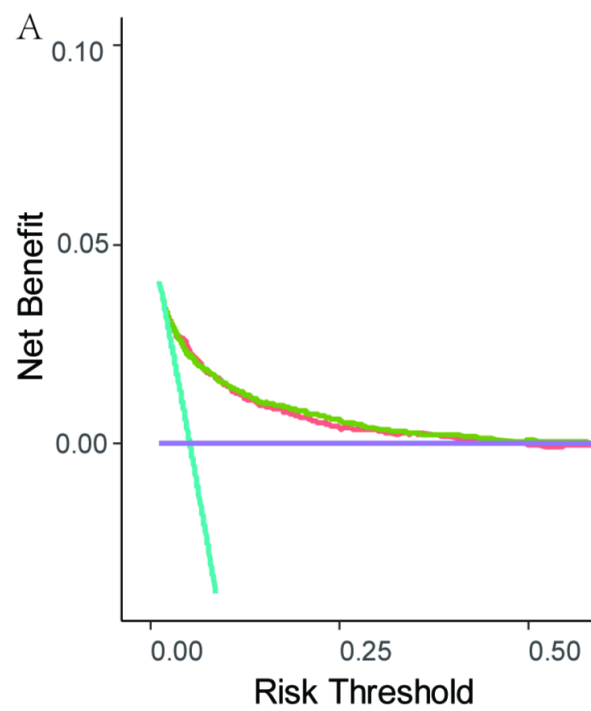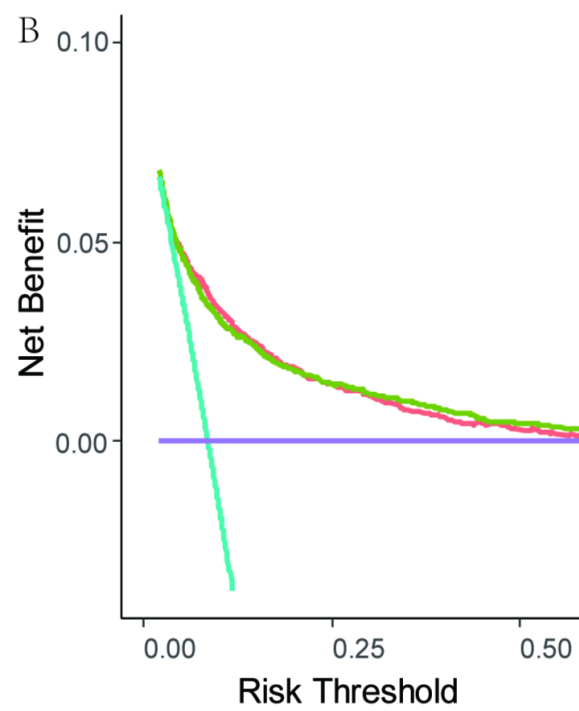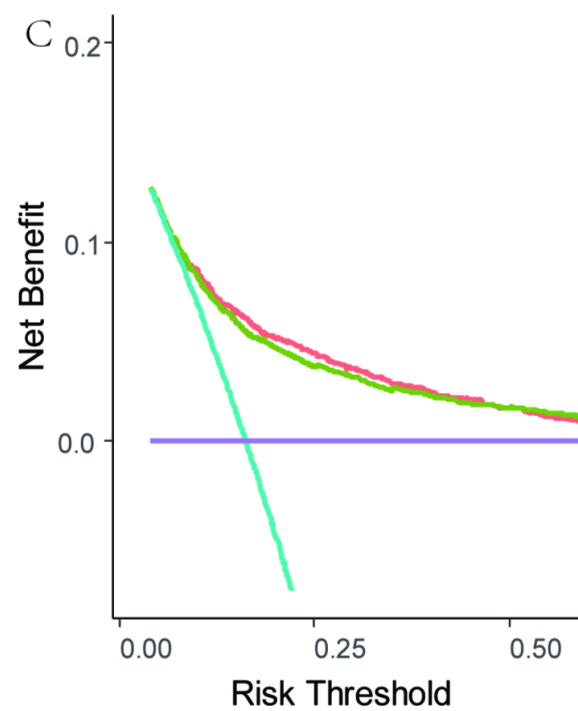

Validate  
Model  
All  
None

Supplement: Supplemental Information 7 [file peerj-13-19823-s007.pdf]

A

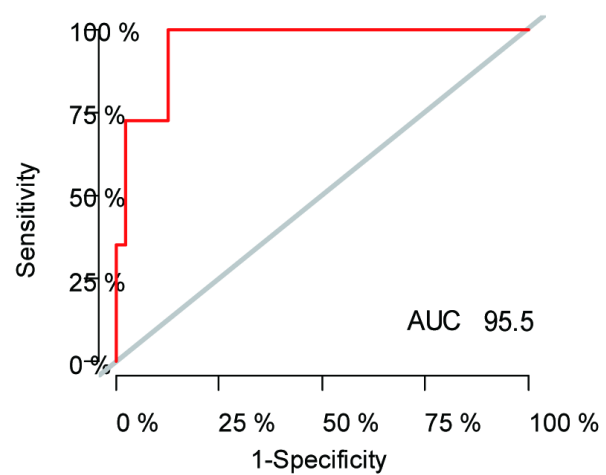

B

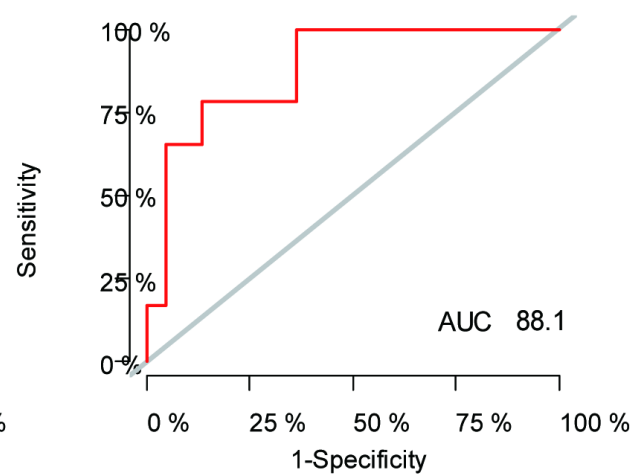

C

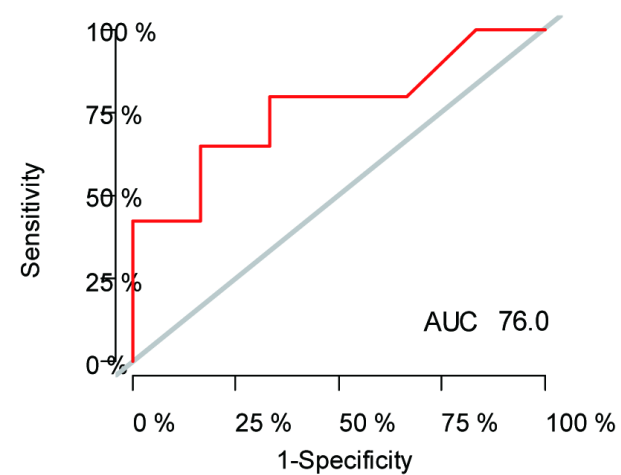

Supplement: Supplemental Information 8 [file peerj-13-19823-s008.pdf]
